# Dietary resveratrol does not delay engraftment, sensitize to vincristine or inhibit growth of high-risk acute lymphoblastic leukemia cells in NOD/SCID mice

**DOI:** 10.3892/ijo.2012.1650

**Published:** 2012-10-04

**Authors:** SUSAN J. ZUNINO, DAVID H. STORMS, JOHN W. NEWMAN, THERESA L. PEDERSEN, CARL L. KEEN, JONATHAN M. DUCORE

**Affiliations:** 1 United States Department of Agriculture, Agricultural Research Service, Western Human Nutrition Research Center, Davis, CA;; 2 Department of Nutrition, University of California Davis, Davis, CA 95616;; 3 Department of Pediatrics, Section of Hematology/Oncology, University of California School of Medicine, Sacramento, CA 95817, USA

**Keywords:** acute lymphoblastic leukemia, dietary resveratrol, NOD/SCID mice

## Abstract

Acute lymphoblastic leukemia (ALL) with translocation t(4;11) is a high-risk leukemia found in 60–85% of infants with ALL and is often refractory to conventional chemotherapeutics after relapse. To evaluate the efficacy of dietary resveratrol *in vivo*, 5-week-old NOD.CB17-Prkdcscid/J mice were fed a control diet or a diet containing 0.2% w/w resveratrol. After 3 weeks of dietary treatment, mice were engrafted with the human t(4;11) ALL line SEM by tail vein injection. Engraftment was monitored by evaluating the presence of human CD19^+^ cells in peripheral blood using flow cytometry. Relative to control diet, dietary resveratrol did not delay the engraftment of the leukemia cells. To determine if dietary resveratrol could increase efficacy of a chemotherapeutic agent, vincristine was injected intraperitoneally into leukemic mice fed the control or supplemented diet. Survival curves and monitoring the percentage of human leukemia cells in peripheral blood showed that resveratrol did not inhibit leukemia cell growth or influence the activity of vincristine. Mass spectrometric analysis of mouse serum revealed that the majority of resveratrol was present as glucuronidated and sulfated metabolites. These data do not support the concept that dietary resveratrol has potential as a preventative agent against the growth of high-risk t(4;11) ALL.

## Introduction

Resveratrol (3,5,4′-trihydroxy-*trans*-stilbene) is a plant polyphenol that is present in grapes, red wine, blueberries, mulberries, and cranberries (1). *In vitro* studies have shown that resveratrol can inhibit proliferation and induce apoptosis in different types of cancer cells (1). *In vivo*, resveratrol has been reported to be an effective agent against breast, esophageal, lung, and colon cancers in animal models (2–6). Furthermore, resveratrol has been reported to sensitize neuroblastoma, glioblastoma, breast carcinoma, prostate carcinoma, leukemia, and pancreatic carcinoma cells to the actions of multiple traditional chemotherapeutic agents *in vitro*(7). At a molecular level, resveratrol has been reported to act as an antioxidant, inhibit transcription factor and kinase activation required for cell growth, and inhibit cell cycle progression (1,8–10).

High-risk acute lymphoblastic leukemia (ALL) with chromosomal translocation t(4;11) has a poor prognosis for patients. A significant problem that limits survival of patients with t(4;11) ALL is the relapse of chemotherapy-resistant leukemia (11,12). The t(4;11) ALL is a pre-B cell leukemia found in 60–85% of infants with ALL. Several cell lines have been established from patients with t(4;11) ALL and these lines have been used *in vitro* to evaluate novel therapeutic agents (13–15). The nonobese diabetic x severe combined immunodeficient (NOD/SCID) mouse model has been useful for evaluating different chemotherapeutic agents against leukemia (16–19). The SCID background has an absence of T and B lymphocyte populations and the NOD background provides reduced natural killer lymphocyte activity and absence of circulating complement that increase engraftment efficiency of human cells. The NOD/SCID mouse model for leukemia mimics the human disease by homing to, and engrafting in, the bone marrow, spleen, and liver. The engraftment in organ sites correlates well with the presence of leukemia cells in the peripheral blood (20).

We have reported that resveratrol, as well as several other plant-derived polyphenols, such as carnosol, curcumin, and quercetin, are effective *in vitro* in inducing apoptotic cell death in t(4;11) and other ALL-derived cell lines (21–23). While resveratrol has been shown to act by stimulating CD95^−^ signaling in some cancer cells, the induction of apoptosis in the t(4;11) ALL lines by resveratrol occurred exclusively by disruption of mitochondrial homeostasis (21). These *in vitro* studies supported the concept that resveratrol has potential as a preventative agent against high-risk leukemia *in vivo.* In the current study, we determined whether dietary resveratrol prevented the engraftment and growth of the t(4;11) ALL cells in NOD/SCID mice. In addition, we tested the hypothesis that it could increase the efficacy of the chemotherapeutic agent vincristine; a standard chemotherapeutic agent used to treat this type of leukemia (24).

## Materials and methods

### Cells and reagents

SEM is a cell line that was established from a patient diagnosed with high-risk pre-B ALL containing the chromosomal translocation t(4;11)(q21;q23) (13). The cells were grown at 37°C, 5% CO_2_ in RPMI-1640 (Invitrogen, Carlsbad, CA) that was supplemented with 10% fetal bovine serum (Sigma, St. Louis, MO), 50 IU/ml penicillin, 50 mg/l streptomycin, 0.25 mg/l amphotericin B, 1 mmol/l sodium pyruvate, and 2 mmol/l L-glutamine (Invitrogen). For injection into mice, SEM cells were collected and washed 2 times in Dulbecco’s PBS without Ca^2+^ or Mg^+^ (Sigma). The cells were resuspended in PBS at a final concentration of 50×10^6^ cells/ml.

Vincristine sulfate (Sigma) was dissolved in PBS and stored at −20°C. To monitor leukemia burden in the blood, peripheral blood leukocytes were stained with phycoerythrincyanin 7 (PE-Cy7)-conjugated anti-human CD19 and allophycocyanin-Cy7 (APC-Cy7) conjugated anti-mouse CD45 (Becton-Dickinson, San Jose, CA). Trans-resveratrol, tetra-deuterated trans-resveratrol (resveratrol-d4), resveratrol-3-O-D-glucuronide, resveratrol-4′-O-D-glucuronide, and resveratrol-3-O-sulfate, and 1-cyclohexyluriedo-3-dodecanoic acid (CUDA) were purchased from Cayman Chemical Co. (Ann Arbor, MI). Sulfatase from *Aerobacter aerogenes*, β-glucuronidase (Type IX-A) from *Escherichia coli*, formic acid, glycerol, potassium 4-nitrophenyl sulfate, and 4-nitrophenyl β-D-glucuronide were obtained from Sigma. Ammonium hydroxide and LC/MS grades of methanol, acetonitrile, and water were purchased from Fisher Scientific (Fair Lawn, NJ). Normal mouse serum was obtained from United States Biological (Swampscott, MA).

### Diets

Resveratrol (>98% pure) was purchased in bulk from Cayman Chemical Co. Rodent Diet 7013 (Harlan Teklad, Madison, WI), a commercial NIH-31 modified diet, was used as the base diet because it is similar in composition to the diet used by Jackson Laboratory for maintenance of the NOD. CB17-Prkdcscid/J mouse strain. The diets used in the current study were prepared by Harlan Teklad. Diet 1 was the base diet (control) and diet 2 was the base diet containing 0.2% w/w resveratrol. A dietary concentration of 0.2% w/w resveratrol is equivalent to approximately 300 mg/kg body weight/day assuming a 20-g mouse and consumption of 3 g of food/day. This dietary concentration was chosen to both minimize the risk for resveratrol-induced renal toxicity (25), and ensure that reasonable blood concentrations of resveratrol would reach the leukemia cells at engraftment sites. For the addition of the supplement, Rodent Diet 7013 was ground, the resveratrol was added, and the diet was repelleted. The control diet was also ground and repelleted so that the consistency of the food for each group was similar. The diets were γ-irradiated to sterilize and packaged in 2 kg vacuum sealed bags to reduce exposure to air. Diets were stored at −20°C until use and fresh food was given to the mice weekly.

### Mice

Experimental procedures using mice were approved by the University of California, Davis Institutional Animal Care and Use Committee. Female NOD.CB17-Prkdcscid/J mice (5 weeks of age) were purchased from the Jackson Laboratory (Bar Harbor, ME, common name NOD/SCID). Upon arrival, the mice were randomly sorted into 2 groups (n=16 per group) and given the control diet or diet containing 0.2% w/w resveratrol. Mice were given sterilized food and water *ad libitum*. Mice were housed under 12 h light-dark cycle, pathogen-free, temperature controlled conditions in ventilator racks at a University of California, Davis vivarium. Mice were weighed weekly. Weighing of mice and injections of leukemia cells or chemotherapeutic agent were performed in a biosafety cabinet to maintain pathogen-free conditions. Additions of food and water, and cage changes were performed in a laminar flow change-out cabinet. Mice were euthanized by carbon dioxide asphyxiation.

### Leukemia cell engraftment

After receiving the diets for 3 weeks, each mouse was injected with 5×10^6^ SEM cells (100 *μ*l volume) through the tail vein. Approximately 2 weeks after injecting the leukemia cells, blood was collected from the tail artery of each mouse once per week using heparinized Microvette tubes (Sarstedt, Newton, NC). Blood from each mouse (approximately 50 *μ*l) was transferred to a separate 1.5 ml microfuge tube and the red blood cells were lysed using PharmLyse (Becton-Dickinson) according to the manufacturer’s suggestions. The resulting peripheral blood leukocytes (PBLs) were stained with PE-Cy7 conjugated anti-human CD19 and APC-Cy7 conjugated anti-mouse CD45. The cells were incubated with antibodies at room temperature for 20 min, washed in PBS containing 0.1% BSA and 7 mmol/l sodium azide (Sigma), and then fixed in 1% paraformaldehyde (Sigma) before analysis on a FACSCanto™ fluorescence-activated cell sorter (FACS) using FACSDiva™ software (Becton-Dickinson). Analysis of leukocytes was performed using appropriate scatter gates to exclude cellular debris and aggregated cells. The negative control was PBLs isolated from NOD/SCID mice that had not been injected with leukemia cells. The positive control was made by spiking an aliquot of PBLs (isolated from mice without leukemia) with SEM cells. Both the negative and positive control cells were stained with PE-Cy7 conjugated anti-human CD19 and APC-Cy7 conjugated anti-mouse CD45 and used to set the gates for human CD19^+^ cells. Thirty thousand events were collected for each sample. Positive engraftment was defined as 1% or greater human CD19^+^ cells present in the murine PBL population (18,19).

### Treatment with vincristine

All mice were injected intraperitoneally (i.p.) with vincristine at a concentration of 0.5 mg/kg body weight 3 times per week beginning approximately 4.5 weeks after injection of leukemia cells (26). The total volume for each injection of vincristine was approximately 100 *μ*l and was adjusted weekly according to the body weight of each mouse. All animals were fed the control or resveratrol diets during the chemotherapeutic treatment, and the percentage of human leukemia cells was monitored by flow cytometry as described above.

### Liquid chromatography (LC) - tandem mass spectrometry (MS) analysis

Serum samples from NOD/SCID mice (n=5) were prepared at the time of euthanization and stored at −70°C until use. Serum from each mouse was separated into three 25 *μ*l aliquots for enzymatic digestion using a mock control (no enzyme), β-glucuronidase, or sulfatase. The serum samples were processed and analyzed as previously described (27).

### Statistical analysis

Statistical analyses were performed with GraphPad software (GraphPad Software, Inc., San Diego, CA) and data are displayed as arithmetic means ± SEM. Event free survival (EFS) was used for comparisons between the treatment groups, and was defined by overt clinical illness requiring euthanization, that included greater than 20% weight loss, lethargy, severe weakness, or inability to reach food or water for 24 h. The EFS was calculated beginning with the day of injection of the leukemia cells. Kaplan-Meier survival curves were used to determine differences in EFS by log-rank test. Differences in the body weights and percentage of CD19^+^ cells between the dietary groups were analyzed by 2-way ANOVA with Bonferroni posttests. The confidence interval for significant differences was set at 95%.

## Results

### Dietary resveratrol does not inhibit engraftment of leukemia

**Figure 1:**
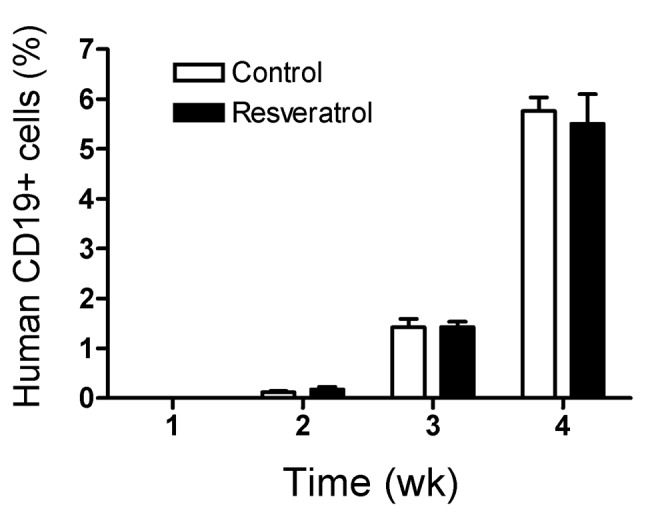
Dietary resveratrol did not inhibit engraftment of t(4;11) ALL cells. Mice were fed control or resveratrol supplemented diets (n=16 per group) for 3 weeks prior to injection of 5×10^6^ SEM leukemia cells into the tail vein. Mice were monitored for engraftment of the leukemia by measuring the percentage of human CD19^+^ cells in the mouse peripheral blood leukocyte (PBL) population by flow cytometry. Engraftment was defined as 1% or greater human CD19^+^ cells present in the murine PBL population. The graph represents the time after injection of the leukemia cell. The bars represent means ± SEM. No difference in the percentage of CD19^+^ cells was observed between the dietary groups (P=0.78).

Mice were fed specialized diets for 3 weeks prior to injecting the leukemia cells to determine if dietary resveratrol delayed engraftment. Engraftment of SEM leukemia cells was 100% in both control and resveratrol fed mice. The rate of engraftment as measured by the percentage of SEM leukemia cells in the PBL population in control and resveratrol fed mice was similar over a 4-week period after injection of the leukemia cells and prior to treatment with vincristine (Fig. 1, P=0.78).

### Dietary resveratrol does not sensitize the leukemia cells to vincristine

**Figure 2:**
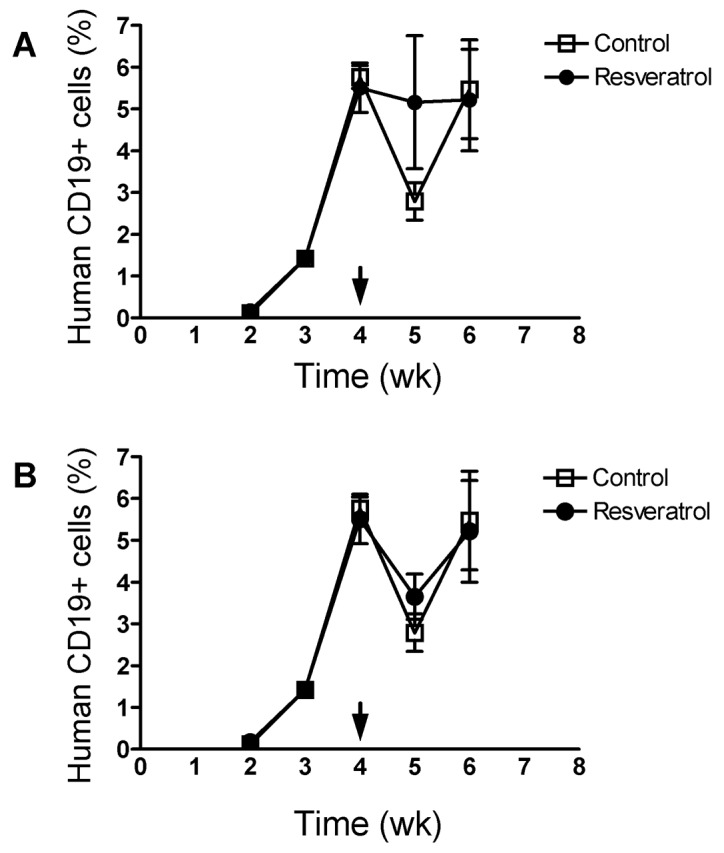
Dietary resveratrol did not sensitize t(4;11) ALL cells to vincristine treatment. Mice were fed control or resveratrol supplemented diets (n=16 per group) for 3 weeks prior to injection of 5×10^6^ SEM leukemia cells into the tail vein. Mice were monitored for engraftment of the leukemia by flow cytometry beginning at 2 weeks after injection of the leukemia cells as described in Fig. 1. Arrows indicate the initiation of vincristine treatment at a dose of 0.5 mg/kg body weight 3 times per week. (A), Represents data for all mice in each group. One mouse in the resveratrol fed group had a leukemia burden of approximately 10% and showed a delayed response to the vincristine treatment, resulting in the large standard error at week 5. (B), Represents data shown in (A) minus the highly engrafted mouse, indicating that vincristine was similarly effective in reducing the leukemic burden in both groups. Data are presented as mean ± SEM. No differences in the percentage of CD19^+^ cells between the dietary groups was observed (2-way ANOVA, P=0.39 using all mice in each group).

Mice were treated with vincristine at week 4 when the mean percentage of human leukemia cells in the mouse PBL population reached approximately 5.8±0.3% for the control group and 5.5±0.6% in the resveratrol fed group (Fig. 2). Mice in both feeding groups initially responded to the vincristine treatment, resulting in a temporary decrease in leukemia burden. One mouse in the resveratrol fed group had a leukemia burden of approximately 10% in the peripheral blood and showed a delayed response to the vincristine treatment, resulting in the large standard error at week 5 (Fig. 2A). Removal of this mouse from the data analysis showed that vincristine treatment produced a similar reduction in the leukemia burden between the two dietary groups (Fig. 2B). Overall, there was no difference in the percentage of human leukemia cells in the mouse PBL population between the mice fed the control or resveratrol diets at weeks 5 and 6 after vincristine treatment (P=0.39, Fig. 2A, all data). After 2 weeks of treatment with vincristine, the leukemia burden began to increase and the mice became clinically ill.

### Dietary resveratrol does not increase survival of leukemic mice

**Figure 3:**
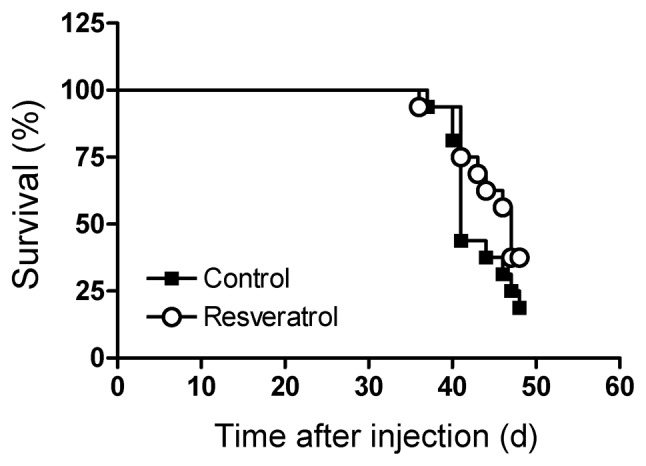
Dietary resveratrol did not increase survival of the leukemic mice compared to the control diet. The mice were fed control or resveratrol supplemented diets 3 weeks prior to injection of the leukemia cells into the tail vein (n=16 per group). Treatment with vincristine was initiated at approximately 28–30 days after injection of the leukemia cells. The mice were euthanized when they became clinically ill. Differences in survival after treatment began were determined by log-rank test. No difference in survival between the dietary groups was observed (P=0.74).

**Figure 4:**
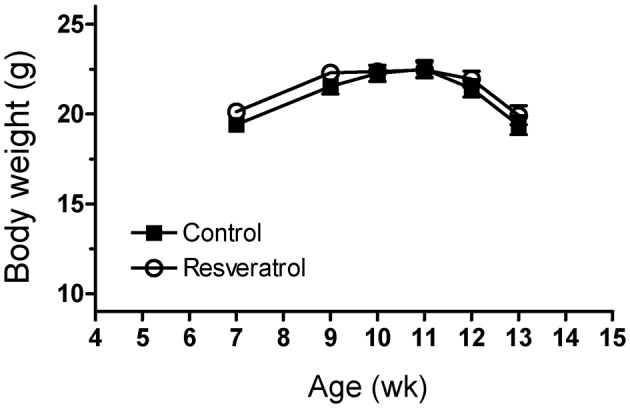
Body weights of the mice fed control or resveratrol supplemented diets were not different. Body weights of the mice were measured weekly. The mice were fed the control or resveratrol diets beginning at the age of 5 weeks and maintained on the diets throughout the experimental period. SEM leukemia cells were injected at the age of 8 weeks. Vincristine treatment was begun at about 12 weeks of age. The data represent means ± SEM. No differences in body weight were observed between the 2 dietary groups (P=0.09).

Mice were fed the control or resveratrol supplemented diets 3 weeks before injection of leukemia cells and throughout the experimental period. Survival was similar between the control and resveratrol supplemented mice (Fig. 3, P=0.74). The mice succumbed to the disease rapidly about 6 weeks after injection of the leukemia cells, and 2 weeks after treatment with vincristine. Mean body weights were similar between the control and resveratrol supplemented mice before and after injection of the leukemia cells at 8 weeks of age (Fig. 4, P=0.09). Body weight loss was evident by 4–5 weeks after the injection of leukemia cells, and the mice continued to lose weight during the vincristine treatment.

### Dietary resveratrol was extensively metabolized to glucuronides and sulfates

**Figure 5:**
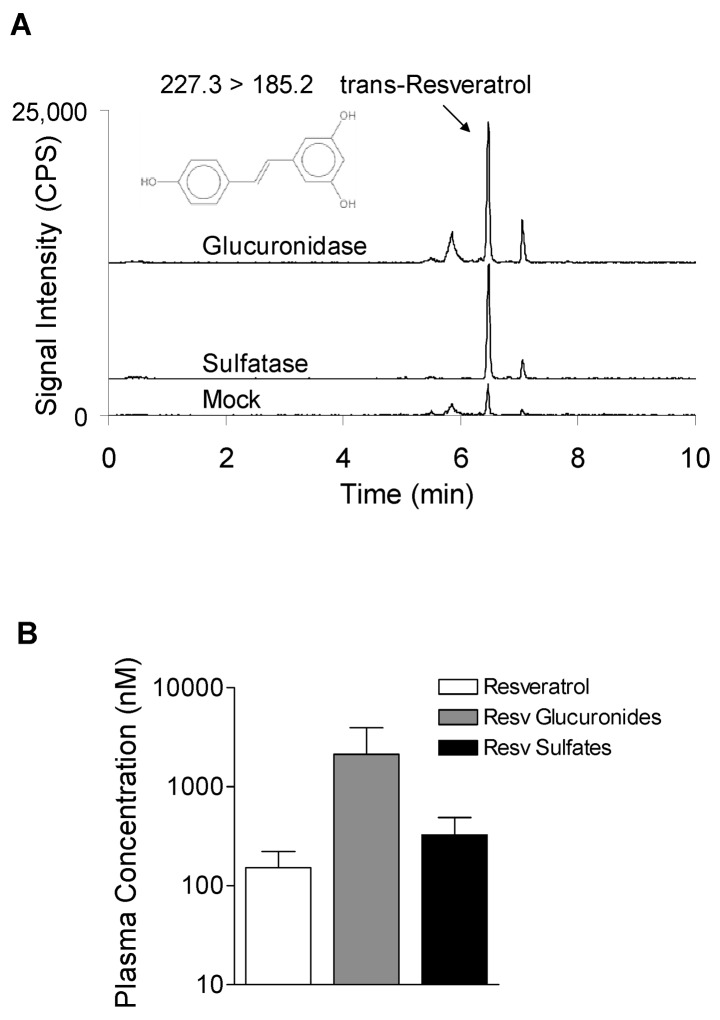
Dietary resveratrol was metabolized to glucuronidated and sulfated forms in leukemic mice. (A), Representative UPLC-MS/MS chromatograms of resveratrol isolated from serum collected from leukemic mice at the time of euthanization shows the metabolic profile of resveratrol after digestion with buffer (mock), β-glucuronidase, or sulfatase. Signal intensities for each mass transition are equivalent in the three panels. Deconjugation reactions increased peak areas of resveratrol relative to mock digestions, demonstrating the generation of glucuronide and sulfate conjugates *in vivo*. (B), Mean plasma concentrations of resveratrol aglycone and metabolites from 5 mice. The percentage of distribution of aglycone: glucuronides: sulfates was 16:57:27. The data represent means ± SEM.

Due to the lack of anti-leukemia activity of resveratrol, the metabolism of resveratrol was evaluated by LC-MS in a subpopulation of surviving mice that were showing signs of clinical illness, but were still mobile and able to reach food (n=5). The mice were euthanized and the serum was either mock digested or digested with β-glucuronidase or sulfatase. The resulting increases in resveratrol aglycone after enzymatic digestion were used to determine the concentrations of glucuronidated and sulfated metabolites. Incubation of serum with either β-glucuronidase or sulfatase increased the peak intensities of extractable resveratrol compared to the mock digested serum (Fig. 5A). The majority of the total resveratrol in the serum was present as metabolites. The mean percentage of resveratrol aglycone was 16±6%, whereas 57±9% and 27±6% of resveratrol was present as monoglucuronides and monosulfates, respectively (Fig. 5B). The 4-nitrophenyl glucuronide and sulfate conjugates (digestion controls) showed greater than 99% deconjugation efficiency. Deuterated resveratrol (surrogate) recoveries were 45±6%, 50±2%, and 62±3% for the mock, β-glucuronidase and sulfatase digestions, respectively.

## Discussion

In human feeding studies, resveratrol has been used at levels up to 5 g/day, with some adverse results, including diarrhea, nausea, and abdominal pain, being reported at doses ranging from 2.5 to 5 g/day (28,29). In a 50–70 kg adult, the dose of 5 g/day corresponds to 71–100 mg/kg body weight/day in humans. Due to the adverse responses in humans, it has been suggested that levels of dietary resveratrol for humans should not exceed 1 g/day. Toxicity studies have been performed in rats with oral administration of 100, 300, and 3000 mg/kg body weight/day and resveratrol was reported to have renal toxicity at 3000 mg/kg body weight (25). In the present study with mice, we chose a dietary concentration of 0.2% w/w resveratrol that is approximately 300 mg/kg body weight/day assuming a 20-mouse and consumption of 3 g of food/day diet. This dietary concentration was used to reduce renal toxicity in the mice, while ensuring there was a high potential of the resveratrol to reach the organs and tissues that harbored the leukemia cells. Despite the high amount (0.2% of the diet) of resveratrol given in the current study, there was no indication that its inclusion in the diet inhibited the engraftment or growth of high-risk t(4;11) leukemia in NOD/SCID mice.

Studies have been performed in both humans and experimental animals to determine the tissue distribution, excretion rates, and general bioavailability of resveratrol after its oral administration. In humans, after 25 mg of ^14^C-labelled resveratrol was given as a single oral dose, the concentration of resveratrol peaked at approximately 500 ng/ml in the plasma (2 *μ*mol/l) at 1 h, with a second peak of approximately 300 ng/ml (1.3 *μ*mol/l) at 6 h (30). In these experiments, there was at least 70% absorption of resveratrol and most of the oral dose was recovered in the urine. Although the plasma levels were low with the single oral dose of 25 mg, absorption was rapid and efficient and the plasma half-life of resveratrol and metabolites was calculated at 9.2 h. The major forms of resveratrol observed in this study were sulfate and glucuronide conjugates. More recently, humans given daily doses of 0.5, 1, 2.5, or 5 g for 29 days showed maximal plasma concentrations of the resveratrol aglycone at 0.19, 0.62, 1.45, and 4.24 *μ*mol/l, respectively (28). However, the time of maximal plasma concentration of resveratrol aglycone in the human study was approximately 1 h and the majority of the resveratrol had been converted to the resveratrol-4′-O-glucuronide, resveratrol-3-O-glucuronide, and resveratrol-3-O-sulfate.

The tissue distribution of orally administered resveratrol has been reported in animal models. In mice, a single oral dose of ^14^C-labelled resveratrol (5 mg/kg body weight) showed distribution in the duodenum, colon, liver, kidney, lung, spleen, heart, brain, and testis by 3 h, with the highest content in the duodenum (31). Longer term experiments utilizing rats showed the presence of resveratrol metabolites in the plasma after an 8-week feeding of 300 mg resveratrol/kg body weight per day (32). The plasma, liver, kidney, urine, and feces contained mainly the sulfated and glucuronidated forms of resveratrol in these rats. By the end of the 8-week feeding study, the concentrations of the different resveratrol conjugates in the plasma ranged from 0.37 to 7.46 mg/l (1.6–32.7 *μ*mol/l). In the present study, only nmol/l levels of resveratrol aglycone were detected, but approximately 2 *μ*mol/l glucuronide metabolites were present in the serum of leukemic NOD/SCID mice. Taken together, the SEM leukemia cells in the target organs (spleen, liver, and bone marrow) would have been mostly exposed to resveratrol metabolites rather than the resveratrol aglycone. Further investigations will be required to evaluate the apoptotic activities of resveratrol metabolites against leukemia cells. However, our data suggest that the leukemia cells were not exposed to high enough levels of resveratrol aglycone and/or metabolites to induce leukemia cell death.

The majority of *in vitro* studies on resveratrol focused on the study of the putative anti-cancer activity of this nutrient have been conducted using nonphysiological *μ*mol/l concentrations of the resveratrol aglycone. We submit that these types of *in vitro* experiments have limited value to elucidate the chemopreventive or therapeutic potential of resveratrol against leukemia *in vivo*. However, we note that resveratrol has been reported to have some efficacy in decreasing tumor burden and metastatic potential of a number of different cancers in rodent models (33). Resveratrol has been reported to inhibit metastasis of lung cancer and melanoma after intraperitoneal injection, and colon carcinoma metastasis after oral administration (34–36). Resveratrol has been reported to sensitize a number of cancers to chemotherapeutic agents *in vitro* and the mechanisms of this sensitization include down-regulation of multi-drug resistant protein expression, modulating the expression of cell survival proteins such as Bcl-2, down-regulating the transcription factor NF-κB, and cell cycle arrest (7). Reversal of doxorubicin resistance in acute myeloid leukemia *in vitro* was reported to be mediated by down-regulation of the multi-drug resistant protein MRP-1, but only at a nonphysiological dose of 50 *μ*mol/l (37). Limited data exist on the chemosensitizing effects of resveratrol *in vivo*. It was recently reported that resveratrol administered daily by gavage (at a dose of 40 mg/kg body weight) for 35 days significantly reduced the size of ectopic pancreatic tumors in nude mice, and that it potentiated the effects of gemcitabine in this model of pancreatic cancer (38). These experiments suggest that resveratrol may have potential as a chemopreventive agent depending upon the type of cancer and/or route of administration, and may be beneficial in the prevention of metastasis.

High-risk t(4;11) ALL is an aggressive leukemia that has a poor prognosis due to development of chemotherapy resistant cells. The t(4;11) ALL line SEM was established from a relapsed patient that had undergone chemotherapy (13). Engraftment of these cells in NOD/SCID mice was achieved rapidly within 2–3 weeks after injection of the leukemia cells. *In vitro*, the SEM cells are responsive to vincristine-induced apoptosis, but upon engraftment into NOD/SCID mice and treatment with vincristine, SEM cells revert to a vincristine-resistant phenotype due in part by an increased expression of the multi-drug resistant protein P-glycoprotein (26). Dietary resveratrol did not sensitize these leukemia cells to vincristine treatment, nor did this dietary nutrient inhibit the rapid engraftment and growth of SEM cells in the NOD/SCID mice. In summary, while dietary resveratrol may have potential as a chemopreventive agent against some cancers, the results presented in the current study suggest that this agent has minimal value with respect to the treatment or prevention of high-risk t(4;11) ALL *in vivo*.
